# Primary treatment of acromegaly with high-dose lanreotide: a case series

**DOI:** 10.1186/1752-1947-4-85

**Published:** 2010-03-08

**Authors:** Christian Wuster, Stefan Both, Uwe Cordes, Wael Omran, Robert Reisch

**Affiliations:** 1 Clinic for Endocrinology, Bahnhofplatz 2, D-55116 Mainz, Germany; 2 Clinic for Radiology, Am Brand 22, D-55116 Mainz, Germany; 3 University Zurich, Department of Neurosurgery, Frauenlinkstr, 10, CH-8091 Zürich, Switzerland

## Abstract

**Introduction:**

The first-line treatment for acromegaly is transsphenoidal surgery. In approximately 50% of patients, however, a cure is not possible with surgery and alternatives are needed. Somatostatin analog therapy is the recommended first-line treatment in patients with such cases. Here we provide the first report of a high-dose lanreotide primary therapy in patients with acromegaly.

**Case presentation:**

Six patients who were not suitable for surgery were given 60 mg of lanreotide (Autogel^®^) every four weeks. All patients were German nationals and Caucasian.

When the response of our patients was unsatisfactory, the dose was increased sequentially to 90 mg every four weeks, 120 mg every four weeks, 120 mg every three weeks and 180 mg every three weeks. Treatment duration was 12 to 24 months. In all cases, the lanreotide dose was 120 mg every 4 weeks or higher. In five of our patients, growth hormone (GH) levels were successfully reduced (in three patients GH <2.5 ng/ml was achieved). Insulin-like growth factor 1 levels were normalized in three patients and decreased in two patients. One patient failed to show a biochemical response to lanreotide therapy or pegvisomant therapy.

Tumor shrinkage or degeneration was observed in the five responding patients. No drug-related adverse events were noted.

**Conclusions:**

These results suggest that lanreotide at high doses of 120 mg every four weeks or more is an effective first-line therapy for patients with acromegaly that surgery alone cannot treat.

## Introduction

Acromegaly, characterized by elevated growth hormone (GH) and insulin-like growth factor 1 (IGF-1) levels, is associated with a range of cardiovascular, respiratory, endocrine, metabolic and compression symptoms, and with an increased cancer risk [1-3]. Some symptoms can be serious and life-threatening. If untreated, acromegaly reduces life expectancy [4,5].

Transsphenoidal surgery is the first-line treatment for acromegaly. However, surgery may be impractical. In approximately 50% of patients, surgery alone is unlikely to control the disease. In cases where there is a low probability of surgical cure, primary treatment with a long-acting somatostatin analog is recommended [6,7].

Two somatostatin analogs are available, such as octreotide (Sandostatin^®^, Novartis) and lanreotide (Autogel^®^, Ipsen). There are considerable clinical data on the first-line use of octreotide (for example, Colao *et al. *[8]). Lanreotide, on the other hand, has been shown to be effective as secondary treatment at starting doses of 30 mg to 120 mg every four weeks [9], but only recently have data been published on the use of lanreotide 90 mg or 120 mg every four weeks as primary therapy [10].

We present six patients with acromegaly who received primary treatment with lanreotide at doses higher than those presented in the literature.

GH, IGF-1 and prolactin levels were measured using chemiluminescent immunometric assays with Immulite 2000 (Siemens Medical Solutions Diagnostics, formerly DPC, Los Angeles, USA). Normal GH levels were defined as 0.5 to 5.0 ng/ml and normal age-adjusted IGF-1 levels were between 81 and 483 ng/ml.

Magnetic resonance tomography was conducted with a 1.5 Tesla-System (Siemens, Erlangen) Type AVANTO, and 1.5 T. MR (Avanto, Siemens). Magnetic resonance imaging (MRI) images were produced using a head matrix array. For pituitary imaging, T2w-TSE sag, T1w-SE sag and coronar before and after contrast medium gadolinium (Gadovist, Bayer Healthcare) were used dynamically. The slice size was 2.2 mm.

The six patients with acromegaly presented in this case series were ineligible for surgery because three of them had macroadenoma that were too large and had parasellar and suprasellar extensions (Patients 1, 5 and 6), while one had an adenoma that was too close to the internal carotid artery (Patient 2). One had cardiac insufficiency stage New York Heart Association (NYHA) III and severe insulin-dependent diabetes mellitus and was thus considered high risk for systemic anesthesia (Patient 3), while one had McCune-Albright syndrome and fibrous dysplasia involving the base of the skull which made transsphenoidal surgery impossible (Patient 4).

All patients were German nationals and Caucasian.

All patients initially received lanreotide by deep subcutaneous injection (Autogel^®^, Ipsen, Paris, France). Each patient was given a single starting dose of 60 mg. After four weeks, their IGF-1 and GH levels were measured. If the IGF-1 levels remained high and the response of our patient to the medication was unsatisfactory, the next dose of lanreotide was increased to 90 mg or 120 mg. If a patient who was receiving treatment with 120 mg, had an unsatisfactory IGF-1 response, the injection interval was reduced to every three weeks. If GH and IGF-1 levels were still elevated in patients receiving lanreotide 120 mg every three weeks, the dose was increased to 180 mg, or 90 mg in each gluteal muscle, every three weeks. For each patient, the dose adjustments were usually made after three injections of lanreotide. In single cases with very high IGF-1-levels, the decision to increase the dose was made earlier based on our previous experience with this treatment.

## Case report 1

A 19-year-old Caucasian woman of German nationality presented to our hospital in October 2006 with persistent visual disorders. A large pituitary tumor was diagnosed by MRI, but was deemed unsuitable for surgical resection due to its parasellar and suprasellar extension.

Acromegaly was confirmed by standard endocrinological examinations. Her GH level was >40 ng/ml and was not suppressed by a standard oral glucose load. Her IGF-1 levels were 631 ng/ml. However, the only clinical sign of acromegaly was hyperhidrosis and there was no evidence of hypopituitarism.

**Figure 1:**
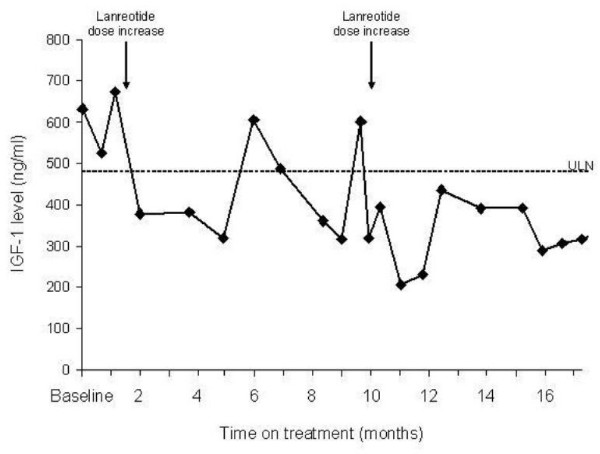
**Insulin-like growth factor 1 values in Case 1 during lanreotide treatment for one year**. Baseline value is pre-treatment value. Lanreotide dose increased from 60 mg to 120 mg every four weeks after one month and to 180 mg every three weeks after 10 months. Upper limit of normal is 483 ng/ml.

Our patient did not respond to the initial dose of lanreotide 60 mg, so the dose was increased to 120 mg every four weeks. Her IGF-1 levels decreased to within the normal range one month after this increase in dosage. She remained relatively stable for 17 months. After 10 months, the dose of lanreotide was increased to 180 mg every three weeks (Figure 1 and Table 1). Hyperhidrosis improved after the normalization of her IGF-1 levels. Her GH levels were substantially reduced after initiating the treatment, but they did not drop to the target of <2.5 ng/ml at any time during the 17 months of treatment (Table 1).

**Table 1:** Growth hormone levels and IGF-1 levels after initiating primary lanreotide treatment in six patients with acromegaly

GH level and IGF-1 level (ng/ml) at different times after starting on lanreotide											
		
S. No	Case	Before treatment	2-4 months	7-9 months	10-12 months	13 months	14-15 months	16 months	17-18 months	20-21 months	23 months
1	GH	>40	6.5	17.2	5.5-12.3		7.3-10.1	9.2	6.6		
	IGF-1	631	378-381	318-488	205-601		390-392	289	317		

2	GH	12	5.6	1.6	1.3-1.8	1.8	1.0-1.2				
	IGF-1	676	482-566	280-345	293-427	411	332				

3	GH	3.9	1.8	2.0	2.5	2.8	2.6	3.1	4.3	4.6	3.6
	IGF-1	621	582	599	630	763-770	695	514	442	565	725

4	GH	5.8**	5.8	3.9	3.6						
	IGF-1	351**	414	395	320						

5	GH	0.6	0.4-0.8	0.5	0.3						
	IGF-1	338	272-301	239-285	212						

6	GH	4.2	1.2-1.6	1.6	1.3				1.3		
	IGF-1	413	285-414	267	286		395		267	260	260

*Patients assessed at different time-points within the range

**Before treatment with lanreotide but after treatment with octreotide for approximately 13 and 27 months for the GH and IGF-1 values, respectively.

**Figure 2:**
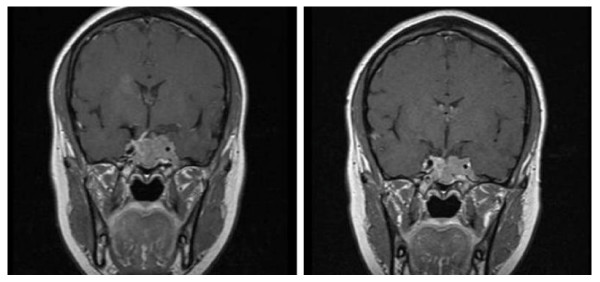
**(A) Magnetic resonance imaging scan showing the tumor size before lanreotide treatment**. (B) Magnetic resonance imaging scan showing a reduction in tumor size after 10 months of lanreotide treatment in Case 1.

The size of her tumor was reduced by approximately 50% (from 4.5 × 4.0 cm to 2.4 × 1.9 cm), 10 months after initiating treatment (Figure 2), but was still not completely resectable by surgery and so no surgical intervention was attempted. Lanreotide treatment continued.

Liver enzyme activities of our patient remained normal throughout, but liver ultrasonography was not performed. Our patient tolerated the treatment well, and she had no complaints of gastrointestinal discomfort.

## Case report 2

A 52-year-old Caucasian woman of German nationality presented to our hospital in January 2007. She was diagnosed with acromegaly when an MRI scan showed a large pituitary tumor that was not suitable for surgical removal because it was too close to the internal carotid artery. Our patient had previously received surgery for bilateral carpal tunnel syndrome and for goitre, had sleep apnoea syndrome, and was experiencing essential tremor. In addition, our patient suffered from a typical enlargement of the nose, ears and lips. Her skin was seborrhoic and she complained of acne. She had depression and was receiving antidepressants.

Her GH levels were 12 ng/ml and were not suppressed by a standard oral glucose load. Her IGF-1 levels were 676 ng/ml. No evidence of hypopituitarism could be found on our patient.

She was given a treatment with lanreotide 60 mg every four weeks and her IGF-1 levels decreased (Table 1). After eight months, however, her IGF-1 levels were still above the upper limit of normal (ULN), and the dose of lanreotide was increased to 120 mg every four weeks. Increases in her IGF-1 levels at 12 months led to a further dose increase to 180 mg every three weeks. In April 2008, (approximately 14 months after initiating treatment), her IGF-1 and GH levels had decreased to within, or close to, the normal range (Table 1), which was at 332 ng/ml for IGF-1 and <2.5 ng/ml for GH.

On publication, MRI images show tumor degeneration with cystic decay within the tumor, and lanreotide treatment continues at 180 mg every four weeks.

Liver enzyme activities of this patient remained normal throughout, but liver ultrasonography was not performed. She tolerated the treatment well and reported no gastrointestinal discomfort.

## Case report 3

A 63-year-old Caucasian woman of German nationality and with a 40-year history of type 1 diabetes and poor glycaemic control with insulin therapy presented to our hospital with a pituitary tumor in April 2006. She had elevated IGF-1 levels of 621 ng/ml, and high to normal GH levels of 3.93 ng/ml. There was no evidence of hypopituitarism. Although she had no overt symptoms of acromegaly, her cardiac function was improved when her GH and IGF-1 levels were reduced as she had less dyspnoea. The management of her diabetes was also more effective (with fewer episodes of severe hypoglycaemia) when her GH and IGF-1 levels were controlled.

In June 2006, she was started on lanreotide 60 mg every four weeks. There was no significant reduction in her IGF-1 levels and the dose of lanreotide was then increased to 120 mg every four weeks. After 11 months of treatment with lanreotide, her IGF-1 and GH levels were largely unchanged from their levels before the treatment (Table 1).

Since she appeared to be insensitive to somatostatin analog treatment and did not consent to a dose increase, the dose was not increased any further. Instead, she was initiated on the GH antagonist pegvisomant (Somavert^®^, Pfizer, UK) at a dosage of 120 mg/day and her lanreotide treatment was stopped. After a further 12 months of this treatment, her GH levels remained high to normal, and her IGF-1 levels remained well above the ULN (Table 1).

No tumor shrinkage was seen in any of her MRI scans.

## Case report 4

Diagnosed with acromegaly and with a suspicion of fibrous dysplasia in November 2004, a 61-year-old Caucasian man of German nationality was noted to have McCune-Albright syndrome (with a G→A mutation in exon 8, codon 201 of the *GNAS-1 *gene) and clinical symptoms that included café au lait marks.

His IGF-1 values were 616 ng/ml (ULN was 212 ng/ml for age-matched controls) at the time of diagnosis, and his GH levels were 8.48 ng/ml and not suppressed by a standard oral glucose load.

Our patient was initially treated with 10 mg of octreotide every four weeks for 27 months. During this time his IGF-1 levels ranged from 329 to 509 ng/ml, and his GH levels ranged from 4.54 to 7.69 ng/ml. Disease control with octreotide was therefore inadequate and our patient was switched to 120 mg of lanreotide every four weeks. After 10 months of treatment with lanreotide, his IGF-1 levels were 320 ng/ml and GH levels were 3.6 ng/ml (Table 1), indicating some improvement in his status. MRI scans showed a tumor size reduction from 8 × 5 mm to 6 × 3 mm.

## Case report 5

In April 2004, a 45-year-old Caucasian man of German nationality presented with a pituitary tumor that measured 3.4 × 3.8 × 3.8 cm, prolactin levels of 530 ng/ml (ULN: 20 ng/ml) and IGF-1 levels of 315 ng/ml (ULN was 212 ng/ml for age-matched controls).

A dopamine agonist, cabergoline 1 mg, was administered to our patient three times per week. The prolactin and IGF-1 levels of our patient was thus normalized (IGF-1 was 188 ng/ml) within four weeks. After one year, an MRI assessment showed that the diameter of the tumor of our patient was reduced to 2.7 × 3.0 × 3.7 cm. By January 2007, and with a continuation of cabergoline doses that suppressed prolactin increases, the IGF-1 levels of our patient had increased to 338 ng/ml. Basal GH levels were 0.56 ng/ml. Therefore, our patient was initiated on a treatment with lanreotide 60 mg every four weeks. Despite reductions in IGF-1 levels to as low as 239 ng/ml after seven months, the lanreotide dose was increased initially to 90 mg every four weeks, and then to 120 mg every four weeks one month later. After 10 months of treatment with lanreotide, our patient's IGF-1 levels had normalized to 212 ng/ml. GH levels remained well below 2.5 ng/ml during the lanreotide therapy. Tumor size was reduced to 2.4 × 2.8 × 3.3 cm.

## Case report 6

A 53-year-old Caucasian man of German nationality presented to our hospital in February 2004 with a pituitary tumor measuring 3.1 × 3.6 cm. Our patient had elevated prolactin values (2940 ng/ml) but normal GH values (0.27 ng/ml) and IGF-1 values (127 ng/ml).

He was initiated on a 1 mg cabergoline treatment three times per week. But it had to be increased after three months to 1 mg/day to normalize his prolactin levels. His IGF-1 levels subsequently increased; reaching 312 ng/ml eight months after presentation and 413 ng/ml 25 months after presentation (ULN was 267 ng/ml for age-matched controls). Similarly, his GH levels increased over time to basal levels of 4.23 ng/ml after 22 months, and were not suppressed by standard oral glucose load. However, 12 months after presentation, the tumor mass had reduced to 2 cm in diameter. In March 2006, he was started on treatment with lanreotide 60 mg every four weeks, and four months later his IGF-1 levels normalized to 285 ng/ml and GH levels was reduced to <2.5 ng/ml (Table 1). Subsequent dose increases of lanreotide to 90 mg every four weeks and then to 120 mg every four weeks maintained his IGF-1 and GH at normal levels. As of publication, the most recent MRI finding shows signs of tumor regression. Cabergoline treatment was continued and unchanged during this time.

No adverse events related to lanreotide treatment were recorded in any of the six patients we described.

## Conclusions

This series of six patients with acromegaly is one of the first specific reports of primary treatment with lanreotide, and the first to report the use of high doses. The six patients were not eligible for surgical removal of their pituitary tumors, and five patients showed a biochemical response to lanreotide treatment, which was given for one to two years. The response of our patients to lanreotide treatment occurred within one to two months, but required dose increases to 120 mg every four weeks, while two patients subsequently required 180 mg every three weeks to achieve or maintain their initial response. Furthermore, the five patients that responded also had evidence of tumor shrinkage or degeneration while receiving lanreotide.

The goals of treatment for acromegaly are to reduce GH levels to <2.5 ng/ml, normalize IGF-1 levels, and/or control tumor mass [6,7]. Based on these goals, lanreotide was proven to be a successful first-line therapy in five of these six patients, with the exception of Case 3, as this patient did not respond to high-dose lanreotide or to pegvisomant.

The other unique aspect of this case series is the high dose of lanreotide (180 mg every three weeks) that was given to two of our patients. This high dose of lanreotide has now been given to one patient for approximately six months and to another for approximately three months with no unexpected adverse events. The use of such high doses of lanreotide has not been previously published, and our experience suggests that this dose is well-tolerated, at least in the short-term, and may be useful for patients showing an attenuation of response to lanreotide doses of 120 mg every four weeks.

This report confirms the efficacy and tolerability of lanreotide in the primary treatment of acromegaly. Further data are required regarding the use of lanreotide in this setting, as well as to identify the potential clinical risks and benefits of high-dose lanreotide.

## Consent

Written informed consent was obtained from all patients for publication of this case report and accompanying image. A copy of the written consent is available for review by the Editor-in-Chief of this journal.

## Competing interests

The authors declare that they have no competing interests.

## Authors' contributions

CW collated the information on our patients from the case notes, structured the manuscript, reviewed the literature and defined the content of the discussion. SB made the MRIs, analyzed and interpreted them, and input into the discussion. UW and WO consulted with our patients, and input into the discussion. RR reanalyzed the MRIs and defined operation indications/schedules. All authors have read and approved the final manuscript.
